# The extracellular loop of Man-PTS subunit IID is responsible for the sensitivity of *Lactococcus garvieae* to garvicins A, B and C

**DOI:** 10.1038/s41598-018-34087-2

**Published:** 2018-10-25

**Authors:** Aleksandra Tymoszewska, Dzung B. Diep, Tamara Aleksandrzak-Piekarczyk

**Affiliations:** 1 Institute of Biochemistry and Biophysics, Polish Academy of Sciences (IBB PAS), Pawińskiego 5a, 02-106, Warsaw, Poland; 2 Faculty of Chemistry, Biotechnology and Food Science, Norwegian University of Life Sciences, Ås, Norway

## Abstract

Mannose phosphotransferase system (Man-PTS) serves as a receptor for several bacteriocins in sensitive bacterial cells, namely subclass IIa bacteriocins (pediocin-like; pediocins) and subclass IId ones - lactococcin A (LcnA), lactococcin B (LcnB) and garvicin Q (GarQ). Here, to identify the receptor for three other narrow-spectrum subclass IId bacteriocins - garvicins A, B and C (GarA-C) *Lactococcus garvieae* mutants resistant to bacteriocins were generated and sequenced to look for mutations responsible for resistance. Spontaneous mutants had their whole genome sequenced while in mutants obtained by integration of pGhost9::IS*S1* regions flanking the integration site were sequenced. For both types of mutants mutations were found in genes encoding Man-PTS components IIC and IID indicating that Man-PTS likely serves as the receptor for these bacteriocins as well. This was subsequently confirmed by deletion of the *man-PTS* operon in the bacteriocin-sensitive *L*. *garvieae* IBB3403, which resulted in resistant cells, and by heterologous expression of appropriate *man-PTS* genes in the resistant *Lactococcus lactis* strains, which resulted in sensitive cells. GarA, GarB, GarC and other Man-PTS-targeting bacteriocins differ in the amino acid sequence and activity spectrum, suggesting that they interact with the receptor through distinct binding patterns. Comparative analyses and genetic studies identified a previously unrecognized extracellular loop of Man-PTS subunit IID (γ+) implicated in the *L*. *garvieae* sensitivity to the bacteriocins studied here. Additionally, individual amino acids localized mostly in the sugar channel-forming transmembrane parts of subunit IIC or in the extracellular parts of IID likely involved in the interaction with each bacteriocin were specified. Finally, template-based 3D models of Man-PTS subunits IIC and IID were built to allow a deeper insight into the Man-PTS structure and functioning.

## Introduction

Mannose phosphotransferase system (Man-PTS) is a major phosphoenolpyruvate (PEP)-dependent sugar transporting system for mannose uptake and its concurrent phosphorylation in *Firmicutes* and *Gammaproteobacteria*^1^. It has a fairly broad substrate specificity as, besides mannose, it can also transport glucose, fructose, glucosamine, N-acetylglucosamine and galactosamine^2^. It is a multi-component system, composed of general PTS proteins, enzyme I and HPr (phosphoryl group donors for different PTS permeases), and enzyme II, which is a permease specific for the carbohydrates listed above. Enzyme II consists of the cytoplasmatic subunits IIA and IIB and the membrane subunits IIC and IID. The intracellular components transfer the phosphoryl group from PEP to the incoming sugar substrate while the membrane components form a sugar-specific binding site and translocation channel^1^. It has been proposed that in addition to its basic transport-related function, Man-PTS can also regulate a variety of intracellular processes, including metabolism, mutagenesis and gene expression^3^. Moreover, Man-PTS is also a target for several antimicrobial agents, such as bacteriocins^4^, and bacteriophage lambda^5^. Importantly, Man-PTS is considered as convenient drug target as it is absent in eukaryotic cells^6^.

Early studies indicating that Man-PTS may serve as a receptor for bacteriocins based on the observation that mutants resistant to some subclass IIa bacteriocins (pediocin-like bacteriocins; pediocins) exhibited no or low level of expression of Man-PTS-encoding genes^7–10^. Also heterologous expression of the Man-PTS-encoding genes in a resistant strain caused sensitivity to pediocins^11^. Nonetheless, a definitive conclusion that Man-PTS is a bacteriocin receptor came from a study by Diep *et al*.^4^. Using immunoprecipitation, they also showed that several subclass IIa bacteriocins and two subclass IId bacteriocins - lactococcin A (LcnA) and lactococcin B (LcnB) (LcnA-like bacteriocins; lactococcins) interact with the components of Man-PTS and that for self-protection, an immunity protein directly binds the receptor and the bacteriocin in a tripartite complex (receptor:bacteriocin:immunity). Recently, another subclass IId bacteriocin, garvicin Q (GarQ), was shown to employ Man-PTS IIC and IID components as target^12^.

Amongst the three Man-PTS phylogenetic groups, only one (group I) can serve as a receptor for class II bacteriocins^13^. This group is characterized by the presence of three distinct regions termed α, β and γ. Region α is localized in the N-terminal part of subunit IIC and, depending on the host sensitivity, it contains a conserved GGQGxxG or GG[D/K]FxxxG sequence, where x indicates any amino acid. Region β is localized in the C-terminal part of subunit IIC, is rich in glycine and contains a conserved DP[I/L/V]GDI[I/L][D/E/N]xY sequence. Region γ is localized in subunit IID and contains a sequence of 35–40 amino acids which is absent in the IID components from phylogenetic groups II and III.

The distinct features of the α, β and γ regions of the phylogenetic group I Man-PTS play a key role in the host sensitivity to bacteriocins. Subclass IIa bacteriocins are known to have a broad antimicrobial spectrum being active against bacteria from the genera *Carnobacterium*, *Clostridium*, *Enterococcus*, *Lactobacillus*, *Leuconostoc*, *Listeria*, *Pediococcus* and *Streptococcus*. They are most active against *Listeria* and *Enterococcus* spp. that harbor the *mptACD* operon encoding Man-PTS containing regions α, β and γ. On the other hand, LcnA-like bacteriocins have an extremely narrow activity spectrum as they target only *Lactococcus lactis* strains in which Man-PTS encoded by the *ptnABCD* operon lacks the α region^6,14^. The susceptibility to pediocins depends on the presence of region α, whereas unspecified regions of both IIC and IID subunits from lactococcal Man-PTS are essential for the specific recognition by lactococcins^6,14^. Interestingly, GarQ is active against both listerial and lactococcal species including *Lactococcus garvieae* that harbors the *manABCD* operon encoding Man-PTS and is resistant to pediocin- and LcnA-like bacteriocins. It has been proposed that the interaction of GarQ with its receptor may involve a few amino acids located in the N-terminal part and the extracellular loop (region γ) of subunit IID and in the transmembrane region of subunit IIC^12^.

The three subclass IId bacteriocins investigated in this study are encoded by plasmids from a human blood isolate *L*. *garvieae* 21881^15^. Garvicin A (GarA) is encoded by pGL5 together with genes responsible for immunity and secretion. It has a narrow activity spectrum limited only to other *L*. *garvieae* strains, including many animal and human pathogenic strains^15^. The other two bacteriocins (accession no. WP_014386584.1 and WP_014386275.1), here named Garvicin B (GarB) and Garvicin C (GarC), are encoded respectively by pGL2 and pGL1 plasmids and, unlike GarA, they are not expressed in the host strain probably due to a lack of genes required for their secretion. The precursors of GarA, GarB and GarC contain leader peptides of 20 or 21 amino acids with a typical double-glycine motif, whereas their mature peptides are 43 (GarA) or 51 (GarB and GarC) amino acids long. The GarA prepeptide shows 50% and 42.9% identity with the GarB and GarC prepeptides, respectively, while mature GarA is 42.3% and 19.6% identical with mature GarB and GarC peptides^15^.

In the present study we identified Man-PTS as a receptor for the three bacteriocins GarA, GarB and GarC. These bacteriocins use distinct bacteriocin - receptor binding patterns when compared to each other and to the Man-PTS-targeting bacteriocins characterized earlier. Among the specific amino acids from distinct regions of *L*. *garvieae* Man-PTS IICD engaged in the bacteriocin – receptor interaction, those from the IID extracellular loop (region γ+) seem to be the most important for the interaction with all three garvicins. The constructed 3D models of Man-PTS IICD indicated transmembrane localization of subunit IIC and monotopic localization of IID suggesting, respectively, entry and docking receptor functions for these subunits.

## Materials and Methods

### Bacterial strains, plasmids and culture conditions

The bacterial strains and plasmids used in this study are listed in Supplementary Table S1. Indicator strains, *L*. *garvieae* IBB3403-derived strains with random mutations, deletion or complementation of the *manABCD* operon, *L*. *lactis* IL1403-derived strains and *L*. *lactis* NZ9000-derived strains were grown in brain heart infusion (BHI) medium (Oxoid, Hampshire, UK) at 30 °C. *L*. *garvieae* IBB3403-derived strains with missense mutations were grown in chemically defined medium (CDM)^16^ supplemented with 1% mannose (man-CDM) at 30 °C. When appropriate, erythromycin (Ery) and/or chloramphenicol (Cam) were added to the concentration of 5 µg/ml each. *Escherichia coli* EC1000-derived strains were grown in Luria-Bertani (LB) medium (Becton, Dickinson and Company, East Rutherford, NJ, USA) at 37 °C. When appropriate erythromycin, chloramphenicol or ampicillin (Amp) were added to 75 µg/ml, 20 µg/ml or 100 µg/ml, respectively. Transcription of the *manABCD* operon cloned in pNZ8037 was induced by the addition of nisin to the concentration between 10 and 50 ng/ml. Soft agar (soft BHI-agar, soft man-CDM-agar) and agar plates (BHI-agar, man-CDM-agar) were prepared by adding agar (Merck, Darmstadt, Germany) to 0.75% and 1.5%, respectively.

### Bacteriocin preparation

Lyophilized bacteriocins with a purity of over 95% for GarA and over 90% for GarB and GarC were synthesized by a commercial service (PepMic, Suzhou, China). Before use, the peptides were dissolved at 1 mg/ml in 0.1% trifluoroacetic acid (TFA) (Sigma, Darmstadt, Germany).

### Inhibitory spectrum assay and selection of resistant mutants

The activity spectrum of bacteriocins was determined and resistant mutants with random or missense mutations within *manABCD* operon were obtained as described before^12^.

### DNA isolation and manipulation

PCR products were purified using Wizard® SV Gel and PCR Clean-Up System (Promega, Fitchburg, WI, USA). Plasmids and genomic DNA were isolated using, respectively, PureYield^TM^ Plasmid Mini-prep System (Promega, Fitchburg, WI, USA) and Genomic Mini Kit (A&A Biotechnology, Gdynia, Poland). Samples for genome sequencing were prepared with Nextera XT DNA Sample Preparation Kit, Nextera XT Indexing Kit and PhiX Control V3 Kit (Illumina, San Diego, CA, USA) according to the manufacturer’s instructions. Miseq Sequencer (Illumina, San Diego, CA, USA) was used for sequencing. For data analysis DNAStar SeqMan Gen program (DNAStar, Madison, WI, USA) was used. Samples for *manCD* sequencing were prepared by PCR with *manC*for/rev and *manD*for/rev primers (Supplementary Table S1). *In silico* translation was performed with the translate tool on the ExPasy online server^17^ (https://www.expasy.org/). Amino acid sequences were compared using the MultAlin software^18^ (http://multalin.toulouse.inra.fr/multalin/) or Clustal Omega software at the EMBL-EBI online server^19^ (https://www.ebi.ac.uk/services). Conserved domains were identify using CD-search online service at the NCBI Conserved Domain Database^20–23^ (http://www.ncbi.nlm.nih.gov/Structure/cdd/cdd.shtml). Prediction of transmembrane protein regions was done with the HMMTOP server^24,25^ (http://www.enzim.hu/hmmtop/). Signal peptide were predicted using SignalP 4.1 online server^26^ (http://www.cbs.dtu.dk/services/SignalP/). Predicted topology of Man-PTS subunits IIC and IID was visualized with Protter^27^ (http://wlab.ethz.ch/protter/). Template-based 3D models of GarA, GarB, GarC and Man-PTS subunits IIC and IID were built with the I-TASSER web service^28–30^ (https://zhanglab.ccmb.med.umich.edu/I-TASSER/). For GarA, GarB and GarC, respectively CDI toxin-immunity protein complex from *E*. *coli* (PDB ID 5HKQA), fragment of FlgE, the hook protein from *Campylobacter jejuni* (PDB ID 5AZ4A) and carbonyl sulfide hydrolase from *Thiobacillus thioparus* (PDB ID 3VQJA) were used as a templates of the highest significance. For IIC and IID, cation-bound Multidrug and Toxin Compound Extrusion transporter from *Vibrio cholerae* (PDB ID 3MKTA) was used as a template of the highest significance. Orientation of the 3D models in the membrane was predicted with the PPM web server^31^ (http://opm.phar.umich.edu). The 3D models were visualized using the PyMOL Molecular Graphics System, Version 2.0 (Schrödinger, LLC; https://pymol.org/2/).

### Non-specific mutagenesis by plasmid integration

Plasmid pGhost9::IS*S1* was transformed into *L*. *garvieae* IBB3403 cells by electroporation and chromosomal DNA was randomly mutagenized by its integration as described by Maguin *et al*.^32^, with minor modifications including the use of BHI medium, 1000-fold dilution and incubation at 30 °C for 150 min and then 37 °C for 150 min. Aliquots of 1, 2 and 5 ml of the mutagenized *L*. *garvieae* IBB3404:pGh9::IS*S1* culture were centrifuged, resuspended in 100 μl of BHI medium and plated onto BHI-agar plates with Ery (5 µg/ml) and GarA (7 µg/ml) preheated to 37 °C and grown for 3 days at 37 °C. Mutant colonies were collected and their level of sensitivity to GarA was estimated using microtiter plates with two-fold bacteriocin dilutions. Next, the DNA rescue cloning procedure was applied as described previously^32^. Briefly, total DNA isolated from the erythromycin- and GarA-resistant pGhost9::IS*S1* integration mutants was digested with EcoRI or HindIII (Thermo Fisher Scientific, Waltham, MA, USA), self-ligated with T4 DNA ligase (Thermo Fisher Scientific, Waltham, MA, USA) and used to transform an *E*. *coli repA*^+^ strain (EC1000) since pGhost::IS*S1* due to its temperature-sensitive replicon (*repA*^−^) is unable to replicate at 37 °C. Clones containing pGhost9::IS*S1* with flanking chromosomal DNA fragments (left - EcoRI or right - HindIII) were selected on LB plates containing Ery. Rescued fragments were sequenced directly from the pGhost9::IS*S1* plasmid with pairs of primers pIS*S1Eco*/pGh9 or pIS*S1Hind*/uni (Supplementary Table S1). The sequences obtained were analysed and compared with the *L*. *garvieae* genome sequence using BLAST network service (NCBI; https://blast.ncbi.nlm.nih.gov) and standard parameters.

### Construction of manABCD deletion mutants

The mutants were created by a double crossover between pGhost9 plasmids harboring DNA fragments flanking the *manABCD* operon or its selected genes and the chromosomal region containing these DNA fragments. DNA fragments flanking the entire *manABCD* operon, *manCD*, *manC* or *manD* genes were created by amplification with primers pairs *manABCD*UPfor/rev and *manABCD*DNfor/rev, *manCD*UPfor/rev and *manABCD*DNfor/rev, *manCD*UPfor/rev and *manC*DNfor/rev or *manD*UPfor/rev and *manABCD*DNfor/rev, respectively (Supplementary Table S1). To each UPrev and DNfor primer the EcoRI restriction site was added. After amplification, PCR products were purified, digested with EcoRI and ligated with T4 DNA ligase, which resulted in DNA fragments containing joined upstream and downstream DNA regions of the *manABCD* gene(s) to be deleted. An additional PCR was performed using suitable UPfor and DNrev primers and the amplified product was ligated with pGEM-T Easy vector (Promega, Fitchburg, WI, USA) by TA cloning and then cloned into pGhost9 using the ApaI and NotI (Thermo Fisher Scientific, Waltham, MA, USA). Overnight *L*. *garvieae* IBB3403 cultures harboring the above pGhost9 derivatives diluted 10^3^-fold in BHI medium with Ery (5 µg/ml). Homologous recombination was enforced by incubation at 30 °C for 1.5 h and at 37 °C for 2.5 h. Integrants were selected at 37 °C on BHI-agar plates containing Ery (5 µg/ml). Excision from the chromosome and removing of the integration vector from the *L*. *garvieae* IBB3403 strains was performed by culturing the integrants in the absence of antibiotic for at least 100 generations at 30 °C. The genetic structure of the resulting deletion strains was confirmed by colony PCR with *manABCD*for/rev primers (Supplementary Table S1), sequencing of the DNA region containing the deleted genes and determination of sensitivity to erythromycin.

### Construction of Man-PTS complementing plasmids

In order to complement the deletion of the *manABCD* operon or its selected genes, two-plasmid nisin-controlled gene expression system (NICE) with pNZ9530 and pNZ8037 plasmids was used^33,34^. Amplification of the entire *manABCD* operon, *manCD*, *manC* or *manD* genes was performed using primer pairs c*omplman*for and c*omplman*rev, c*omplCD*for and c*omplman*rev, c*omplCD*for and c*omplC*rev or c*omplD*for and c*omplman*rev, respectively (Supplementary Table S1). Resulting DNA fragments were purified, digested with NcoI and XhoI and ligated with pNZ8037 plasmid. Obtained constructs were expressed in *L*. *garvieae* 548a, *L*. *lactis* B464 and/or *L*. *lactis* NZ9000. Additional complementing plasmid was created by amplification of the pNZ8037 plasmid harboring *manCD* genes with γ+ for/rev primers (Supplementary Table S1). Obtained construct was expressed in *L*. *lactis* B464.

## Results

### GarA, GarB and GarC have a narrow activity spectrum

GarA activity has been examined earlier against strains from the genera *Bordetella*, *Carnobacterium*, *Enterococcus*, *Lactobacillus*, *Lactococcus*, *Listeria*, *Pediococcus*, *Salmonella*, *Staphylococcus* and *Streptococcus* and found to be limited to *L*. *garvieae*^15^. In this study we used an expanded set of strains to determine the activity spectra of GarB and GarC and reexamine it for GarA. In addition to the above, we included several strains from the genera *Bacillus*, *Campylobacter*, *Candida*, *Leuconostoc* and *Pseudomonas* (Supplementary Table S2). GarA and GarB showed a narrow spectrum of antimicrobial activity, being highly active only against *L*. *garvieae* strains; at a high concentration (1 mg/ml), GarA exhibited minimal activity against *L*. *lactis* strains and some strains from the genera *Carnobacterium*, *Enterococcus*, *Lactobacillus*, *Leuconostoc* and *Pediococcus* (Supplementary Table S2). GarC exhibited a slightly wider spectrum, being potent against all *Lactococcus* spp. (*L*. *garvieae*, *L*. *lactis* and *L*. *raffinolactis*) strains and, to a lesser extent, also against some strains from the genera *Lactobacillus* and *Leuconostoc* (Supplementary Table S2).

### Mutants resistant to GarA-C contain mutations in the manABCD operon

To identify the receptor(s) for GarA, GarB, and GarC we obtained two groups of bacteriocin-resistant *L*. *garvieae* IBB3403 mutants. The first group comprised spontaneously arising resistant strains selected against increasing bacteriocin concentration following a standard procedure^12,35–37^. Fifteen GarA-resistant mutants were obtained (MS1011-MS1014, MS1016-MS1018, MS1027-MS1029, MS1031-MS1033, MS1035 and MS1036; Supplementary Table S1) by exposing wild-type *L*. *garvieae* IBB3403 to 12 μg/ml on BHI-agar plates. The obtained mutants were over 1024-fold less sensitive to GarA than the parental strain and also showed full resistance to GarB and GarC. Remarkably, subsequent genome sequencing of seven randomly selected spontaneous mutants (MS1027-MS1029, MS1032, MS1033, MS1035, MS1036) revealed the presence of single mutations exclusively in the *manABCD* operon encoding the Man-PTS system. Direct sequencing of the *manABCD* operon in the remaining eight mutants unveiled single mutations in *manC* (MS1012, MS1018 and MS1033) or in *manD* (MS1011, MS1013, MS1014, MS1016, MS1017, MS1027-MS1029, MS1031, MS1032, MS1035 and MS1036). All the mutations were nonsense or frameshift ones, leading to premature termination of the *manC* or *manD* ORFs (Table 1). Notably, none of the resistant mutants had mutations in the *manAB* gene (Table 1), suggesting that the encoded polypeptide is not directly involved in the sensitivity to the bacteriocins.

**Table 1:** *L*. *garvieae* IBB3403 mutants resistant to GarA, GarB, GarC and GarQ.

Mutant strain	Mutation	Amino acid change	Resistance to [fold-increased relative to WT]				Position of affected amino acid(s)	Growth on mannose
			GarA	GarB	GarC	GarQ		
GarA-resistant *L*. *garvieae* IBB3403 mutants								
MS1	pGhost9::IS*S1* integration at 714 in *manC*	Leu238 → fs	>1024	>64	>128	>1024	extracellular	−
MS2, MS3	pGhost9::IS*S1* integration at 449 in *manD*	Val150 → fs	>1024	>64	>128	>1024	transmembrane	−
MS1011	G283 → T in *manD*	Glu95 → STOP	>1024	>64	>128	>1024	extracellular	−
MS1012	C163 → T in *manC*	Gln55 → STOP	>1024	>64	>128	>1024	intracellular	−
MS1013, MS1017	C401 → A in *manD*	Ser134 → STOP	>1024	>64	>128	>1024	transmembrane	−
MS1014, MS1028, cMS1032, MS1036	AAT66 → GTA in *manD*	Met23 → STOP	>1024	>64	>128	>1024	extracellular	−
MS1016	G689 → A in *manD*	Trp230 → STOP	>1024	>64	>128	>1024	extracellular	−
MS1018	C359 → A in *manC*	Ser120 → STOP	>1024	>64	>128	>1024	transmembrane	−
MS1027	insertion in *manD* at position 273 C	Ala91 → fs	>1024	>64	>128	>1024	extracellular	−
MS1029, MS1035	G259 → T in *manD*	Glu87 → STOP	>1024	>64	>128	>1024	extracellular	−
MS1031	insertion in *manD* at position 937 A	Gln313 → fs	>1024	>64	>128	>1024	extracellular	−
MS1033	A247 → GG in *manC*	Met83 → fs	>1024	>64	>128	>1024	transmembrane	−
LGA2	T176 → C in *manC*	Leu59 → Pro	32	32	32	128	intracellular	+
LGA3, LGA5	G155 → T in *manC*	Gly52 → Val	4	64	16	4	transmembrane	+
LGA6, LGA13	GTT insertion at 570–571 in *manC*	Val insertion at 190–191	8	32	64	4	transmembrane	+
LGA10, LGA14	C173 → A in *manD*	Ala58 → Asp	2	4	4	2	extracellular (N-terminus)	+
GarB-resistant *L*. *garvieae* IBB3403 mutants								
LGB1-LGB5, LGB7-LGB10, LGA8	TTGTTGGTAACGGTGTTGCTGGT788 → GTGTTGCTGGT in *manD*	262AsnValValGly265 deletion	2	>64	8	4	extracellular (region γ+)	+
LGB6	C937 → A in *manD*	Gln313 → Lys	0	>64	2	2	extracellular	+
GarC-resistant *L*. *garvieae* IBB3403 mutants								
LGC1, LGC12, LGC19	G102 → A in *manD*	Met34 → Ile	0	0	>128	0	extracellular (N-terminus)	+
LGC3-LGC6, LGC9, LGC11, LGC20	C607 → T in *manD*	Arg203 → Cys	0	>64	>128	2	extracellular	+
LGC8	T676 → G in *manD*	Tyr226 → Asp	8	>64	>128	16	extracellular (region γ)	+
LGC13	C317 → T in *manC*	Ala106 → Val	2	>64	>128	4	transmembrane	+
LGC15	C398 → T in *manD*, G943 → A in *manD*	Ala133 → Val, Val315 → Met	2	>64	>128	4	transmembrane, extracellular	+
LGC16	G608 → T in *manD*	Arg203 → Leu	0	>64	>128	0	extracellular	+
LGC18	G563 → T in *manC*	Gly188 → Val	2	>64	>128	4	transmembrane	+
GarQ-resistant *L*. *garvieae* IBB3403 mutants								
PW202	C299 → A in *manC*	Pro100 → His	4	0	8	>1024	transmembrane	+
PW203	C368 → T in *manD*	Thr123 → Ile	0	>64	0	>1024	extracellular (N-terminus)	+
PW204	C331 → T in *manD*	Pro111 → Ser	0	>64	0	>1024	extracellular (N-terminus)	+
LGN1	G902 → T in *manD*	Gly301 → Val	8	>64	32	>1024	extracellular (region γ)	+
LGN2	C398 → T in *manD*	Ala133 → Val	0	4	16	16	transmembrane	+
LGN4	G767 → T in *manD*	Trp256 → Leu	0	4	8	16	extracellular (region γ+)	+
LGN9	C305 → T in *manC*	Ala102 → Val	0	>64	8	16	transmembrane	+

The second type of mutants were obtained by random integration of the pGhost9::IS*S1*^32^. To identify the mutated sites in the resulting GarA-resistant mutants, we sequenced regions flanking the integration site of the plasmid. Three integration mutants were obtained (*L*. *garvieae* MS1–MS3; Supplementary Table S1) exhibiting MIC_GarA_ values over 1024-fold higher than the wild-type *L*. *garvieae* IBB3403 (0.024 µg/ml). The mutants showed full resistance to GarB and GarC, indicating cross-resistance between these bacteriocins. The DNA regions flanking the plasmid integration site in these mutants were successfully cloned, sequenced, and the obtained sequences were compared with the wild-type *L*. *garvieae* IBB3403 genome. Also in these strains, the pGhost9:IS*S1* insertions occurred at two locations in the operon encoding the Man-PTS system, one between positions 714–715 in the *manC* gene (*L*. *garvieae* MS1) and one between positions 449–450 in the *manD* gene (*L*. *garvieae* MS2 and MS3; Table 1; Supplementary Table S1).

### Man-PTS subunits IIC and IID are necessary for GarA-C activity

**Figure 1:**
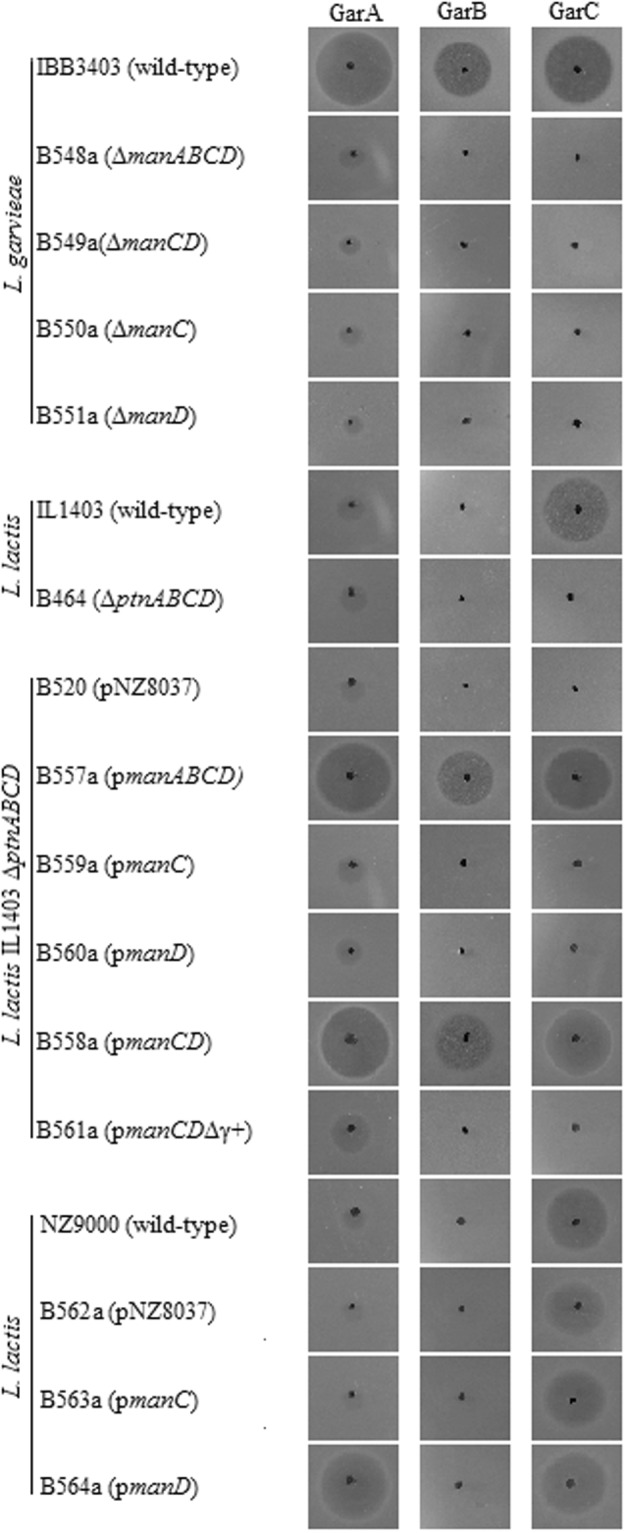
Sensitivity of *L*. *garvieae* IBB3403 and *L*. *lactis* IL1403 strains to GarA, GarB and GarC.

In order to examine whether the resistance to GarA, GarB and GarC was directly related to the *man* operon and not to any additional mutations, we deleted the operon in the sensitive *L*. *garvieae* IBB3403. Additionally, to evaluate the role of individual Man-PTS membrane subunits in the sensitivity to bacteriocins, we deleted the *manC* and/or *manD* genes. All these deletion mutants (*L*. *garvieae* B548a, B549a, B550a and B551a; Table S1 in the supplementary file) had MIC_GarA_ values over 1024-fold higher than the wild-type strain, and, all exhibited full resistance to GarB and GarC (Fig. 1). As GarA and GarC but not GarB exhibited also activity against *L*. *lactis* strains, we performed additional studies considering this species only with two first bacteriocins. Tested *L*. *lactis* B464 strain, which has the mannose-specific PTS operon (*ptnABCD*) deleted^4^, was insensitive to GarC but not to GarA (Fig. 1), indicating that in this species the Man-PTS system is required for sensitivity to GarC only.

To assess the individual role of Man-PTS subunits in mediating bacteriocin sensitivity, we introduced respective genes in different combinations into the *manABCD-*deleted *L*. *garvieae* B548a strain. Since high expression of genes encoding membrane proteins is often toxic to the host cell, we applied a two-plasmid nisin-controlled gene expression (NICE) system allowing for strict control of protein production^33,34^. We used pNZ8037 plasmid with a nisin-responsive promoter for cloning and expressed it in *manABCD-*deleted *L*. *garvieae* B552a with the pNZ950 plasmid carrying *nisK* and *nisR* genes encoding membrane-located histidine kinase NisK and intracellular response regulator NisR, respectively. Unfortunately, even using this system we were unable to express the cloned genes due to plasmid instability. To overcome this problem, we employed the *L*. *lactis* B464 strain with *ptnABCD* deletion, which had been shown to support expression of *man-PTS* genes from the NICE system^4^. Introduction of *manC* or *manD* genes individually into *L*. *lactis* B464 (to give respectively *L*. *lactis* B559a or B560a; Table S1 in the supplementary file) had no impact on the sensitivity of *L*. *lactis* B464 to GarA, GarB and GarC. Only introduction of the entire *manABCD* operon or the *manCD* genes together (respectively *L*. *lactis* B557a and B558a; Table S1 in the supplementary file) produced clones sensitive to the bacteriocins (Fig. 1). Altogether, the results showed unequivocally that both the IIC and IID subunits of *L*. *garvieae* Man-PTS are required and sufficient for sensitivity to GarA-C.

### GarA-C differ from other Man-PTS-binding bacteriocins

**Figure 2:**
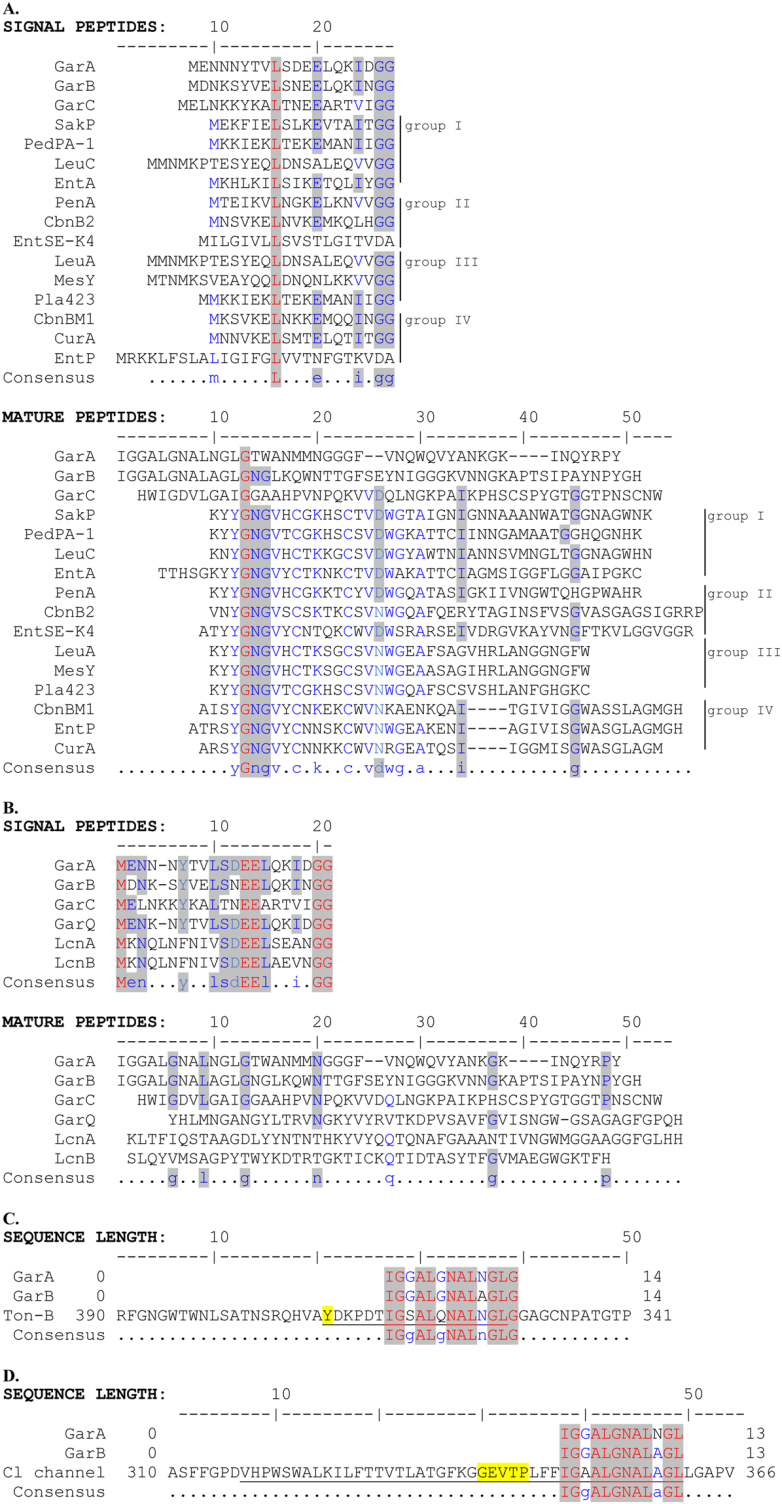
Multiple sequence alignment of the amino acid sequences of signal peptides and mature peptides of garvicin A, B, C and other Man-PTS-targeting bacteriocins: (**A**) subclass IIa bacteriocins, (**B**) subclass IId bacteriocins or channel-forming proteins: (**C**) Ton-B dependent receptor (Ton-B) and (**D**) voltage-gated chloride channel (Cl channel). Consensus amino acids are in red, partial consensus amino acids are in blue. Amino acids conserved between GarA, GarB, GarC and subclass IIa, subclass IId bacteriocins or transport proteins are highlighted by grey background. Conserved amino acids residues and underlined. Amino acids probably involved in the ligand-binding (**C**) and formation of Cl^−^ selectivity filter (**D**) are highlighted by yellow background. The UniProt accession numbers are P38580 for carnobacteriocin B2 (CbnB2), P38579 for carnobacteriocin BM1 (CbnBM1), P0A311 for curvacin A (CurA), Q47784 for enterocin A (EntA), O30434 for enterocin P (EntP), C9B989 for enterocin SE-K4 (EntSE-K4), H2B2W4 for GarA, H2AM33 for GarB, H2AM30 for GarC, H6U5Y1 for GarQ, P0A313 for LcnA, P35518 for LcnB, P34034 for leucocin A (LeuA), H2EST2 for leucocin C (LeuC), P38577 for mesentericin Y105 (MesY), P29430 for pediocin PA-1 (PedPA-1), M1GJ1 for penocin A (PenA), Q93FV7 for plantaricin 423 (Pla423) and P35618 for sakacin P (SakP).

**Figure 3:**
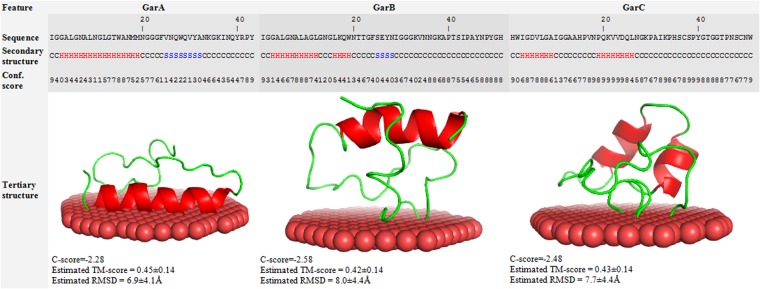
Predicted secondary and tertiary structures of GarA, GarB and GarC. H, S and C indicate helix, strand and coil, respectively. Confidence score range from 0 to 9 and represents certainty of the secondary structure prediction. C- and TM-scores estimate global accuracy of the 3D structure model. C-score in the range from −5 to 2 and TM-score > −1.5 indicates a model with correct global topology. Root mean square distance (RMSD) is the average distance of pairs of residues between model and template.

Pediocins, lactococcins and GarQ have previously been shown to use Man-PTS as a receptor^4,12^. To assess the relatedness of GarA, GarB and GarC to those bacteriocins, we compared their amino acid sequences. Neither the leader nor the mature peptides of GarA, GarB and GarC were similar to those of subclass IIa bacteriocins (pediocins)^38^ (Fig. 2A). In contrast, the leader peptides of GarA-C were highly similar to each other and also to the leader peptides of other subclass IId bacteriocins (LcnA, LcnB and GarQ) on their entire length. In the mature peptides similarity was very weak among GarA-C and virtually absent with LcnA, LcnB and GarQ (Fig. 2B), except for 13 N-terminal amino acids 92% identical between GarA and GarB. The predicted secondary structures of GarA-C comprised one long (17 aa; GarA) or two shorter (GarB and GarC) α-helices at the N-terminus and unstructured C-terminal parts (Fig. 3). However, a comparison of their template-based 3D structure models revealed little overall similarity (Fig. 3). Moreover, GarA was predicted to interact with the membrane through its long α-helix parallel to the membrane surface, while the interaction of GarB and GarC was predicted to occur through the unstructured fragments (Fig. 3). These results suggest that initial electrostatic interaction between cationic bacteriocin and negatively charged bacterial cell membrane may differ between GarA-C. This interaction between cell membrane and N-terminal α-helix of GarA may be also responsible for the observed bacteriocin minimal activity against *L*. *lactis* strains and some *Carnobacterium*, *Enterococcus*, *Lactobacillus*, *Leuconostoc* and *Pediococcus* strains.

In order to get a deeper insight into the phylogeny of the garvicins and thus their possible mechanism of action, we performed a wide homology search using the NCBI BLAST network service. BLASTp showed that the N-terminal 13 amino acids of GarA and GarB are respectively 85% and 77% identical to a region of TonB-dependent receptor (accession no. WP_091746097.1). The similarity region is a part of the ligand-gated channel and is localized adjacent to the conserved amino acid residues responsible for the ligand binding (Fig. 2C). The same N-terminal 12 amino acids of GarA and GarB are also respectively 83% and 92% identical to a region of the voltage-gated chloride channel protein (accession no. WP_058617448.1). This region is a part of the transmembrane α-helix adjacent to the conserved amino acid residues responsible for chloride ion binding and taking part in the formation of the membrane channel^39,40^ (Fig. 2D). Similarity between garvicins and intermembrane fragments of channel forming proteins suggests that studies bacteriocins may act by forming a pore that leads to uncontrolled leakage of intracellular solutes and consequent cell death.

### GarA-C target a specific extracellular loop of subunit IID

**Figure 4:**
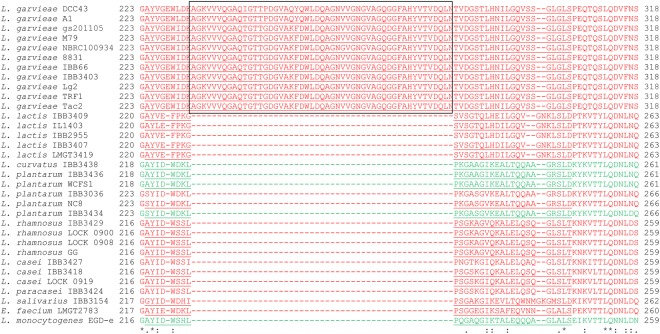
Multiple sequence alignment of the amino acid sequences of Man-PTS subunits IID from various bacterial species. Extracellular and intracellular regions are marked in red and green, respectively. Region γ is underlined, region γ+ is boxed. Asterisks, double dots and single dots indicate fully, strongly and weakly conserved residues, respectively. The accession numbers are WP_003134976.1 for *L*. *garvieae* DCC43, WP_081164986.1 for *L*. *garvieae* A1, WP_046400791.1 for *L*. *garvieae* Lg-ilsanpaik-gs201105, WP_042218841.1 for *L*. *garvieae* NBRC100934, WP_017368922.1 for *L*. *garvieae* 8831, ETD04540.1 for *L*. *garvieae* TRF1, WP_074750596.1 for *L*. *garvieae* M79, WP_004258904.1 for *L*. *garvieae* Lg2, WP_017370845.1 for *L*. *garvieae* Tac2, NP_267864.1 for *L*. *lactis* IL1403, YP_004888576.1 for *L*. *plantarum* WCFS1, WP_003640910.1 for *L*. *plantarum* NC8, WP_005685034.1 for *L*. *rhamnosus* LOCK 0900 *L*. *rhamnosus* LOCK 0908 and *L*. *rhamnosus* GG, WP_003568236.1 for *L*. *casei* LOCK 0919, NP_463631.1 for *L*. *monocytogenes* EGD-e.

In order to identify the Man-PTSs features responsible for sensitivity to GarA, GarB and GarC, we compared the amino acid sequences of IIC and IID form sensitive and resistant species. The IIC subunits from *L*. *garvieae* strains showed high similarity to those from the other species, especially *L*. *lactis* (data not shown). Also the IID subunits showed high conservation, but with notable exception of a stretch of 51 amino acids present only in *L*. *garvieae* (Fig. 4). As the additional sequence lies in the extracellular region γ^13^, we propose to call the part of extended region γ, which contains the additional 51 amino acids, as region γ+. A homology search showed that region γ+ is unique to *L*. *garvieae* strains. To test whether it may indeed be responsible for the sensitivity of *L*. *garvieae* strains to garvicins A, B and C, we deleted in frame an internal part of *manD* encoding the additional 51 amino acids (γ+) from the garvicin-sensitive *L*. *lactis* strain B558a, carrying *manCD in trans* and with *ptnABCD* deleted, which gave in *L*. *lactis* B561a (supplementary Table S1). Deletion of γ+ conferred partial resistance to GarA and full resistance to GarB and GarC (Fig. 1), without affecting the mannose transport function. This indicates that region γ+ of subunit IID is indispensable for sensitivity to GarB and GarC, and, to a lesser extent, also GarA.

### Hybrid Man-PTS system can serve as GarA receptor

As the protein sequences of the Man-PTS IIC subunits of *L*. *garvieae* and *L*. *lactis* were highly similar, we tested whether they can substitute each other to confer sensitivity to GarA and GarB. Therefore, we expressed *L*. *garvieae* genes *manC* or *manD* in the GarA- and GarB-resistant *L*. *lactis* NZ9000, which harbors the entire *ptn* operon and *nisRK* genes on its chromosome. *L*. *lactis* carrying *manC* (B563a) remained resistant to GarA and GarB, while *L*. *lactis* carrying *manD* (B564a) became sensitive to GarA but remained resistant to GarB (Fig. 1; Table S1 in the supplementary file). This indicates that a hybrid Man-PTS system consisting of *L*. *lactis* IIC and *L*. *garvieae* IID subunits can serve as a GarA receptor and that the site required for the antimicrobial activity of GarA is localized in the *L*. *garvieae* IID subunit. On the other hand, both IIC and IID subunits from *L*. *garvieae* are required for GarB activity.

### GarA-C cause mutations at distinct sites of Man-PTS

The results presented above suggested that GarA, GarB and GarC may interact with Man-PTS by using different binding patterns. To pinpoint specific amino acids on IICD necessary for the Man-PTS interaction with individual garvicins, we determined the sensitivity to GarA, GarB and GarC of GarQ-resistant and *man*^+^ (mannose-positive phenotype; Table 1) *L*. *garvieae* IBB3403 missense mutants harboring single amino acid substitutions in the transmembrane domains of IIC (PW202 and LGN9) and transmembrane or extracellular domains of IID (PW203, PW204, LGN1, LGN2, LGN4) obtained in a previous study^12^ (Supplementary Table S1). In that study we established that spontaneous mutants with nonsense or frameshift mutations in the *manABCD* operon are unable to ferment mannose (*man*^−^ phenotype; Table 1) due to premature termination or a change of Man-PTS structure depriving it of its sugar-transporting function. On the other hand, maintained ability to utilize mannose (*man*^+^ phenotype) by missense mutants indicates that the mutations, although leading to GarQ resistance, had no negative effect on the transmembrane structure of Man-PTS and that the amino acid(s) changed were likely involved in the GarQ - receptor interaction. The mutations turned out to affect also the sensitivity to other garvicins. Crucially for the present study, the effects were different for different garvicins. Thus, the PW202 and LGN1 mutants had, respectively, a 4-fold and 8-fold higher MIC value towards GarA than the wild-type strain, while the other mutants remained fully sensitive to GarA (Table 1). In the case of GarB, the MIC was at least 64-fold higher for PW203, PW204 and LGN1, and only 4-fold higher for LGN2 and LGN4, than that of the wild type (0.78 µg/ml). PW202 remained fully sensitive to GarB (Table 1). For GarC the MIC values were 32-fold for LGN1, 16-fold for LGN2, and 8-fold higher than the wild type (0.39 µg/ml) for PW202, LGN4 and LGN9. The other mutants remained fully sensitive to GarC (Table 1). These results convincingly show that distinct Man-PTS mutations differently affect its interaction with individual garvicins.

**Figure 5:**
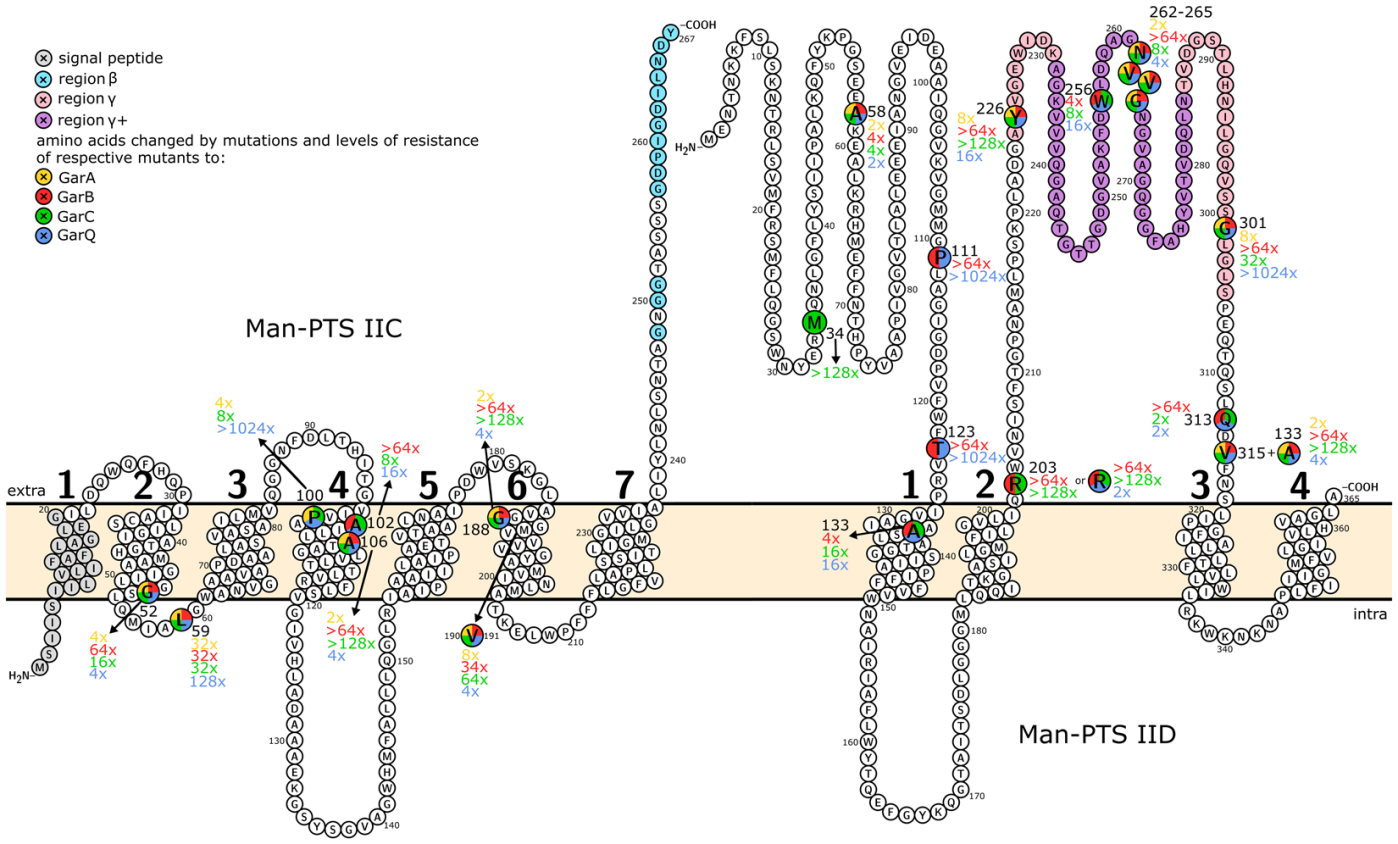
Predicted topology of *L*. *garvieae* IBB3403 Man-PTS subunits IIC and IID.

To identify other Man-PTS regions or specific amino acids targeted by GarA, GarB or GarC we obtained additional spontaneous bacteriocin-resistant mutants with a preserved Man-PTS functionality (*man*^+^ phenotype; Table 1), using the described method^12^. Wild-type *L*. *garvieae* IBB3403 was cultivated on CDM-agar containing mannose as the sole carbon source supplemented with GarA, GarB or GarC. Eight GarA-resistant *man*^+^ mutants were obtained (LGA2, LGA3, LGA5; LGA6, LGA8, LGA10, LGA13 and LGA14; Table S1 in the supplementary file). Sequencing of their *man-PTS* operon revealed five independent mutations. Gly52 → Val substitution (LGA3, LGA5) in the second transmembrane domain of subunit IIC reduced GarA-sensitivity 32-fold, Leu59 → Pro substitution (LGA2) in the intracellular region of IIC – 4-fold and Ala58 → Asp substitution (LGA10, LGA14) in the N-terminus of the IID – 2-fold (Table 1; Fig. 5). Insertion of Val between positions 190 and 191 in the sixth transmembrane domain of IIC (LGA6, LGA13) reduced GarA-sensitivity 8-fold, and deletion of AsnValValGly from positions 262–265 in region γ+ of subunit IID (LGA8) – 2-fold (Table 1; Fig. 5). All these GarA-resistant mutants exhibited also between 4-fold and 64-fold higher resistance to GarB and GarC, and between 2-fold and 128-fold higher resistance to GarQ (Table 1; Fig. 5).

Ten GarB-resistant mutants were obtained (LGB1-LGB10; Table S1 in the supplementary file) containing only two mutations. Deletion of AsnValValGly from subunit IID, the same as that in the GarA-selected mutant (LGA8) clearly represented a hot spot as it was found in nine GarB-resistant mutants (LGB1-LGB5, LGB7-LGB10). This deletion decreased the sensitivity to GarB 64-fold compared with the wild type, 8-fold to GarC, 4-fold to GarQ, and 2-fold to GarA (same as for the LGA8 mutant). The remaining mutant (LGB6) also had a mutation in *manD* resulting in the Gln313 → Lys substitution in the extracellular loop of IID (Fig. 5). It was over 64-fold less sensitive to GarB and 2-fold to GarQ, and was fully sensitive to GarA (Table 1; Fig. 5).

On man-CDM-GarC plates fifteen mutants were obtained (LGC1, LGC3-LGC6, LGC8, LGC9, LGC11-LGC13, LC15, LGC16, LGC18-LGC20) which were over 128-fold less sensitive to GarC than the parental *L*. *garvieae* IBB3403 (Table S1 in the supplementary file). They contained eight mutations, six in *manD* and two in *manC*. In most of the mutants the extracellular loop of the IID was affected by the following substitutions: Arg203 → Cys (LGC3-LGC6, LGC9, LC11, LGC20), Arg203 → Leu (LGC16), Tyr226 → Asp (LGC8), or Val315 → Met (LGC15). Other mutations in IID were in the N-terminal part and in the first transmembrane domain and comprised, respectively, Met34 → Ile (LGC1, LGC12, LGC19) and Ala133 → Val (LGC15) substitutions (Table 1; Fig. 5). Only two mutants had mutations in the fourth and sixth transmembrane domain of subunit IIC: Ala106 → Val (LGC13) and Gly188 → Val (LGC18), respectively (Table 1; Fig. 5). Most of the GarC-resistant mutants were also resistant, to varying degrees, to the other garvicins tested, especially to GarB, for which thirteen strains had the MIC 64-fold higher than the wild type (Table 1).

Altogether, 33 independent resistant mutants were obtained, carrying 13 different mutations (Table 1; Fig. 5). Several features of interest could be observed. The mutants exhibited varying degrees of resistance (between 2-fold and 128-fold higher relative to wild-type) to the selected garvicins and also varying degrees of cross-resistance. Mutations in *manD* gene were significantly more frequent than in *manC* gene, extracellular loop of IID being the most common region affected. Deletion of amino acids 262AsnValValGly265 in the γ+ region was particularly frequent, as it occurred independently ten times. These features clearly confirm our earlier conclusion that γ+ region of subunit IID is a major site of interaction with GarA-C and different regions of subunits IIC and IID are critical for the interactions with different garvicins.

### Amino acids involved in the sensitivity to GarA-C are in the channel of IIC and in the extracellular parts of IID

**Figure 6:**
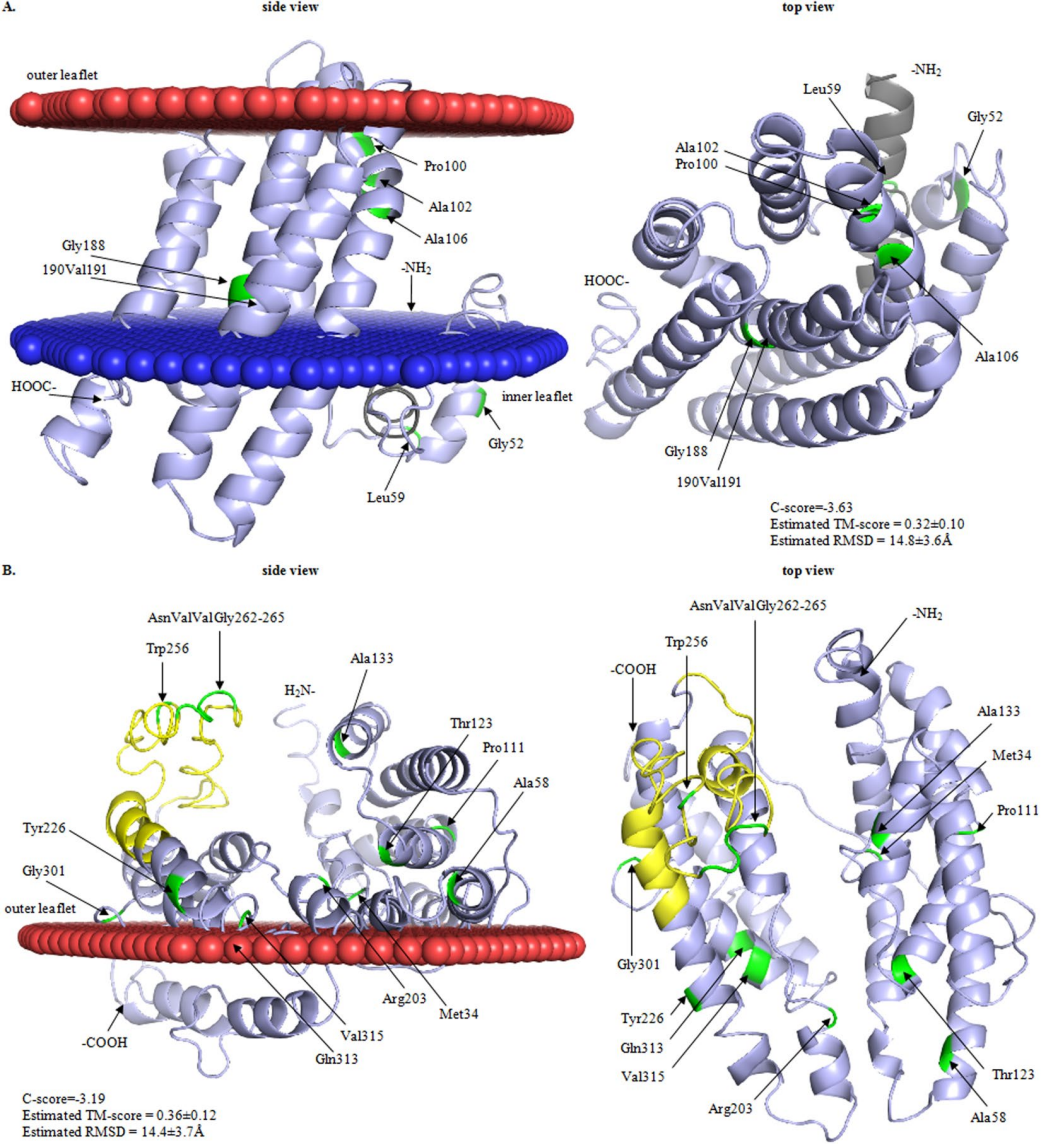
Predicted tertiary structure of *L*. *garvieae* IBB3403 Man-PTS subunits IIC (**A**) and IID (**B**). Amino acids changed by mutations, signal peptide and region γ+ are in green, grey and yellow, respectively. Lipid bilayer is represented by red (outer bilayer) and blue (internal bilayer) layers of spheres. C- and TM-scores estimate global accuracy of the 3D structure model. C-score in the range from −5 to 2 and TM-score > −1.5 indicates a model with correct global topology. RMSD is the average distance of pairs of residues between model and template.

To determine the location of the amino acids altered by the resistance-conferring mutations in the folded IIC and IID subunits we built their template-based 3D models and compared them with the 2D topographic predictions. The two structure predictions coincided with each other fairly well (Figs 5 and 6). In IIC differences were minor and concerned the localization of the N-terminal fragment and the presence of an additional C-terminal transmembrane region. Importantly, the central part of the protein where all the mutations were found had a similar location in the two models (Figs 5 and 6A). Both models predict a transmembrane location of IIC, and the 3D top view exposes a channel for the transport of sugars. The channel is formed by six α-helices and most of the amino acid residues substituted in the garvicin-resistant mutants potentially engaged in garvicin binding (Pro100, Ala102, Ala106 and Gly188) are localized within it (Fig. 6A).

In contrast to IIC, the 3D model of subunit IID does not confirm its transmembrane localization predicted by the topological model but instead indicates that is a monotopic membrane protein only anchored in the outside part of lipid bilayer, that suggests that it can be a first target for bacteriocin binding. Nearly all IID is exposed to the milieu except for amino acids 171–178 and 320–365 (C-terminus), which in the 2D model are part of an intracellular domain and form the third and fourth transmembrane helices, respectively (Figs 5 and 6B). All the amino acids altered by mutations in IID are in its extracellular part (Fig. 6B).

## Discussion

Some *L*. *garvieae* strains are pathogenic and cause lactococcosis in marine or freshwater fish^41^, mastitis in cows^42^, and pneumonia in pigs^43^. Recently, *L*. *garvieae* has also been considered to be a human pathogen as it was isolated from patients with diverticulitis, peritonitis^44^, endocarditis^45^, spondylodiscitis^46^, and acute acalculous cholecystitis^47^. In this study we focused on three bacteriocins, GarA, GarB and GarC, encoded by plasmids from *L*. *garvieae* 21881 isolated from the blood of a patient suffering from septicemia^48^. We confirmed GarA activity against *L*. *garvieae*^15^ and revealed for the first time GarB activity exclusively against *L*. *garvieae* and GarC – against *Lactococcus* spp. All three bacteriocins showed no significant activity against any other lactic acid bacteria tested or diverse pathogenic species, which makes them useful for selective treatment of infections caused by pathogenic *L*. *garvieae* strains.

At present, the most commonly used method for the identification of bacteriocin receptors or other proteins involved in their action, is genome sequencing. It requires generation of spontaneous resistant mutants followed by whole genome sequencing and identification of mutated genes responsible for the reduced sensitivity^12,35–37^. In this study, we also applied an integrative plasmid pGhost9::IS*S1*^32^ to generate bacteriocin-resistant mutants. We have previously used this approach to identify the gene *ccpA*^49^ and several other genes involved in cellobiose and lactose metabolism^50^ in *L*. *lactis* IL1403. However, this approach has not been applied to date to search for genes involved in the sensitivity to bacteriocins (e.g., potential receptor genes). Sequencing of the DNA regions surrounding the pGhost9::IS*S1* integration sites in the genomes of resistant *L*. *garvieae* IBB3403 mutants revealed genes encoding Man-PTS, indicating that it likely is the receptor of the bacteriocins. We confirmed the involvement of these genes by using genome sequencing of spontaneous garvicin-resistant mutants (Table 1). Of the three genes encoding Man-PTS subunits, mutations were found exclusively in *manC* or *manD* and not in *manAB*, indicating that the latter is not required for sensitivity to GarA, GarB or GarC. Those results also showed that pGhost9::IS*S1* integration can be used as a cheaper alternative to whole genome sequencing in searching for bacteriocin receptors.

Deletion of individual *man* genes in *L*. *garvieae* IBB3403 and subsequent complementation studies confirmed an involvement of Man-PTS subunits IIC and IID in the sensitivity to GarA, GarB and GarC. Similarly, deletion of *ptnABCD* in *L*. *lactis* IL1403 confirmed its involvement in the sensitivity to GarC. An attempt to complement the *manABCD* deletion using nisin-inducible pNZ9530 and pNZ8037 plasmids was unsuccessful probably due to incompatibility between them and the native *L*. *garvieae* IBB3403 plasmids. We therefore decided to use the garvicin-resistant, plasmid-free ∆*ptnABCD L*. *lactis* B464^4^ as the host for complementation studies. Introduction of the entire *manABCD* operon resulted in a strain sensitivity to the bacteriocins studied. In parallel, introduction of separately cloned *manC* and *manD* coding for IIC and IID confirmed that both these Man-PTS transmembrane subunits are indispensable for GarA, GarB and GarC activity (Fig. 1).

It has been demonstrated earlier that all subclass IIa bacteriocins and subclass IId LcnA, LcnB and GarQ use Man-PTS as the receptor on target cells^4,12^. Although GarA, GarB and GarC target the same receptor as the other Man-PTS-targeting bacteriocins, a comparison of their activity spectra, amino acid sequences (Fig. 2A,B) and predicted secondary and tertiary structures (Fig. 3) suggested that various bacteriocins may bind differently to their targets. A comparative analysis of the amino acid sequences of subunits IIC and IID from several garvicin-sensitive and garvicin-resistant species revealed high conservation of subunit IIC and the presence of an additional region γ+ unique to IID of *L*. *garvieae* (Fig. 4) likely responsible for the GarA and GarB activity, which is also limited to this species. Moreover, prediction of the IID transmembrane structure showed its external localization. Deletion of the γ+ region resulted in full resistance to GarB and partial resistance to GarA suggesting its key role in the interaction with GarB and a lesser significance in GarA binding. Interestingly, the deletion of the γ+ region also resulted in full resistance to GarC, although, besides *L*. *garvieae*, it is also active against *L*. *lactis*, which lacks region γ+. This suggests that GarC uses different interaction patterns in different species or that the γ+ deletion resulted in a Man-PTS structure change that prevented its binding.

The results discussed above indicated differences in the binding patterns of GarA, GarB and GarC which likely reflected specific interactions between a given bacteriocin and individual amino acids in Man-PTS IICD. Further confirmation of this notion comes from the fact that IIC and IID from *L*. *garvieae* only could serve as a receptor for GarB, whereas GarA recognized the IIC subunit from both *L*. *lactis* and *L*. *garvieae* (Fig. 1). Testing of the GarA, GarB and GarC activity against a panel of *L*. *garvieae* IBB3403 IIC or IID mutants selected as partially resistant to one of the three garvicins (Table 1) allowed the identification of 18 amino acids, six in IIC and twelve in IID, likely involved in specific interactions with the bacteriocins (Fig. 5). Most of those in IIC localized to the sugar-transporting channel and all in IID were extracellular (Fig. 6). The distribution of these amino acids in Man-PTS subunits suggests their diverse functions, probably some directly in the bacteriocin binding and others participating indirectly. The surface location of IID predisposes it to the role of a docking receptor, in which the protruding region γ+ and N-terminal part would serve for the initial contact of a bacteriocin with Man-PTS. The bacteriocin binding could induce IID conformational changes forcing its IIC partner to open the channel. Then, the interior of the open channel of IIC would offer a secondary binding site for the bacteriocin, which would cause a lethal fully open channel structure eventually leading to the cell death. This notion is further supported by the finding that the highly conserved N-terminal parts of garvicins A and B are significantly similar to the transmembrane segments of some bacterial transporters such as TonB and chloride channels (Fig. 2C,D). These proteins take part in the uptake of iron and nickel complexes, vitamin B12 and carbohydrates, and serve as colicin, microcin and bacteriophage receptors (TonB-dependent transporter) or allow passive diffusion of Cl^−^, the Cl^−^/H^+^ exchange, water and salt transport, pH and cell volume regulation, and stabilization of the membrane potential (voltage-gated chloride channel protein)^39,40^. It is feasible that the N-terminal fragments of GarA and GarB could act in a similar manner by docking into the IIC channel to form a stably open pore increasing the membrane permeability. A similar sequence of bacteriocin binding has already been suggested for GarQ^12^, but here the picture becomes more detailed with the support of the modeled structures of IID as the surface docking site and IIC as the entry channel.

The Man-PTS amino acids altered in the mutants with reduced sensitivity to garvicins are apparently of different levels of importance. Some of them were essential for one or two garvicins and caused high resistance levels when mutated, while others had a lesser effect, i.e., they produced low resistance levels when mutated. This indicates a possibility of diverse binding strength between receptor-bacteriocin particular amino acids: an indispensable role in garvicins binding of amino acids, which substitutions led to high resistance levels (from 32x for GarA to 1024x for GarQ) and supporting role of amino acids that substitutions resulted in low resistance of mutants obtained (e.g., only 2–8-fold higher resistance of mutants in comparison with wild-type strain). These observations provide a further clue indicating that individual garvicins use different binding patterns. Nevertheless, one cannot at present exclude the possibility that in fact the altered amino acids are not directly involved in bacteriocin binding but instead are important for the Man-PTS structure, changes of which prevent bacteriocin binding without compromising the mannose transport function (*man*^+^ phenotype of the mutants).

So far, no tertiary structure of a Man-PTS subunit has been reported, which made it difficult to interpret the obtained results in light of the bacteriocin - Man-PTS interactions. We therefore resorted to homology modeling to predict the structures of the two subunits relevant to the present study. Importantly, the overall distribution of transmembrane and extracellular regions corresponded well with the predicted membrane topology of these proteins (Figs 5 and 6). The models confirmed the transmembrane localization of IIC and predicted an unexpected cell-surface localization of subunit IID (Fig. 6). This subunit was earlier proposed to be a transmembrane protein forming a heterotetramer or higher multimer^6^. A cell-surface exposed IID subunit, as predicted here, would greatly facilitate bacteriocin binding via some of the following amino acids: Met34, Ala58, Pro111, Thr123, Ala133, Arg203, Tyr226, Trp256, AsnValValGly262–265, Gly301, Gln313 and Val315, all having an extracellular location and found to be important for bacteriocin sensitivity.

According to the present study, GarA at high concentrations exhibited low activity against *L*. *lactis* and some strains from the genera *Carnobacterium*, *Enterococcus*, *Lactobacillus*, *Leuconostoc* and *Pediococcus*. Also GarC was minimally active against some strains from the genera *Lactobacillus* and *Leuconostoc*. Importantly, a minimal GarA activity was also observed after mutation or deletion of the Man-PTS-encoding genes in both *L*. *garvieae* and *L*. *lactis* (Fig. 1) suggesting that it is not dependent on Man-PTS. As such, it was not the subject of this study. It is possible that at high concentration, after initial unspecific electrostatic interaction between GarA N-terminal α-helix and membrane surface, bacteriocin aggregates and its α-helix penetrates the membrane and form pores without specific receptor. Similarly, it has already been shown that the cyclic bacteriocin garvicin ML exhibits non-specific antimicrobial activity at high concentrations, while at lower concentrations it requires a specific membrane receptor for action^51^. Thus, further studies with different GarA and GarC concentrations are required to evaluate their mode of action at high concentrations^52–69^.

In summary, this study provides a proof-of-principle for a convenient alternative to whole genome sequencing for the identification of genes involved in bacterial sensitivity to bacteriocins. Results obtained using both these methods indicate that in *L*. *garviaeae* Man-PTS subunits IIC and IID act as receptors for GarA, GarB and GarC, three distinct bacteriocins encoded by plasmids of *L*. *garviaeae* 21881 isolated from a clinical case of septicemia. We suggest here that their bactericidal effect, directed mainly against *L*. *garvieae*, relies on garvicin binding to specific amino acids of IICD result in IIC channel opening and cell death. The amino acid residues critical for the garvicin action were identified by analyzing resistant mutants, and their location in the extracellular regions of the surface protein IID and in the transmembrane channel of the IIC permease was determined with the help of predicted topology and 3D structure of the two Man-PTS subunits. Hundreds of bacteriocins have been identified so far with different antimicrobial spectra, which is commonly believed to indicate that they recognize distinct receptors on target cells. Here, however, we provide evidence that the different structures and inhibition spectra of bacteriocins do not necessarily mean that they recognize different receptors. We show that a single receptor, the mannose-specific PTS, can serve as a target for a number of non-homologous bacteriocins with greatly different activity spectra. As we expect that bacteriocins binding Man-PTS constitute a much larger family than the four investigated here, our future studies will focus on a search for other bacteriocins targeting the membrane subunits of Man-PTS. Their detailed investigation will allow us to build a full picture of the bacteriocin - Man-PTS interactions and could eventually justify proposing a separate group of bacteriocins besides the currently recognized IIa-IId.

## Electronic supplementary material

Supplementary Information

## Data Availability

The authors declare that all data generated or analyzed during this study are included in this article.
