# Supplementary material for: The extracellular loop of Man-PTS subunit IID is responsible for the sensitivity of Lactococcus garvieae to garvicins A, B and C
Source: Sci Rep. 2018 Oct 25;8:15790. doi: 10.1038/s41598-018-34087-2 (PMC6202411; doi:10.1038/s41598-018-34087-2)
Supplement: Supplementary file 1 — Supplementary Information [file 41598_2018_34087_MOESM1_ESM.pdf]

The extracellular loop of Man-PTS subunit IID is responsible for the sensitivity of *Lactococcus garvieae* to garvicins A, B and C

ALEKSANDRA TYMOSZEWSKA[1], DZUNG B. DIEP[2] AND TAMARA ALEKSANDRZAK-PIEKARCZYK[1*]

[1]Institute of Biochemistry and Biophysics, Polish Academy of Sciences (IBB PAS), Pawińskiego 5a, 02-106 Warsaw, Poland

[2]Faculty of Chemistry, Biotechnology and Food Science, Norwegian University of Life Sciences, Ås, Norway

*Corresponding author. Mailing address: IBB PAS, Pawińskiego 5a, 02-106 Warsaw, Poland, Phone: (4822) 592 1213, Fax: (4822) 658 4636, E-mail: tamara@ibb.waw.pl

SUPPLEMENTARY MATERIALS

Table S1. Bacterial strains, plasmids and primers used in this study

Strains, plasmids, primers	Description	Source (reference)
Strains		
Bacillus cereus IBB3390	indicator strain	IBB PAS
Bacillus subtilis BSB1	indicator strain	[52]
Campylobacter jejuni 12	indicator strain	[53]
Campylobacter jejuni 480	indicator strain	[54]
Campylobacter jejuni 81176	indicator strain	[55]
Campylobacter coli 23/1	indicator strain	[53]
Candida albicans CAI-4	indicator strain,	[56]
Carnobacterium maltaromaticum IBB3447	indicator strain	IBB PAS
Enterococcus durans IBB3441	indicator strain	IBB PAS
Enterococcus faecalis IBB3439	indicator strain	IBB PAS
Enterococcus faecalis IBB3444	indicator strain	IBB PAS
Enterococcus faecalis LMGT 2003	indicator strain	LMGT NMBU
Enterococcus faecium LMGT 2783	indicator strain	LMGT NMBU
Enterococcus faecium LMGT 2787	indicator strain	LMGT NMBU
Escherichia coli EC1000	host strain, Km^r, *repA*$^+$ derivative of MC1000, carrying a single copy of the pWV01 *repA* gene in *glgB*	[57]
Escherichia coli TG1	host strain, Δ(*hsdMS-mcrB*) 5Δ(*lac-proAB*) *supE thi-1* F'(*traD36 proAB*$^+$*lacI*^{q}ZΔ*M15*)	[58]
Lactobacillus casei IBB3418	indicator strain	IBB PAS
Lactobacillus casei IBB3427	indicator strain	IBB PAS
Lactobacillus casei LOCK 0919	indicator strain	LOCK[59]
Lactobacillus casei/paracasei IBB3423	indicator strain	IBB PAS
Lactobacillus casei/paracasei IBB3425	indicator strain	IBB PAS
Lactobacillus casei/paracasei IBB3426	indicator strain	IBB PAS
Lactobacillus casei/paracasei IBB3428	indicator strain	IBB PAS
Lactobacillus paracasei IBB3424	indicator strain	IBB PAS
Lactobacillus johnsonii IBB3155	indicator strain	IBB PAS
Lactobacillus kunkeei AH1	indicator strain	LMGT NMBU
Lactobacillus kunkeei AH38	indicator strain	LMGT NMBU
Lactobacillus kunkeei AH119	indicator strain	LMGT NMBU
Lactobacillus paraplantarum IBB3438	indicator strain	IBB PAS
Lactobacillus plantarum NC8	indicator strain	LMGT NMBU[60]
Lactobacillus plantarum WCSF1	indicator strain	LMGT NMBU[61]
Lactobacillus plantarum IBB3036	indicator strain	IBB PAS
Lactobacillus plantarum IBB3433	indicator strain	IBB PAS
Lactobacillus plantarum IBB3436	indicator strain	IBB PAS
Lactobacillus plantarum subsp. *plantarum* IBB3434	indicator strain	IBB PAS
Lactobacillus rhamnosus IBB3429	indicator strain	IBB PAS

Lactobacillus rhamnosus LOCK 0900	indicator strain	LOCK[62]
Lactobacillus rhamnosus LOCK 0908	indicator strain	LOCK[63]
Lactobacillus rhamnosus GG	indicator strain	Dicoflor[64]
Lactobacillus salivarius IBB3154	indicator strain	IBB PAS[65]
Lactococcus garvieae IBB3403	indicator strain	IBB PAS
Lactococcus garvieae IBB66	indicator strain	IBB PAS
Lactococcus lactis IBB3404	indicator strain	IBB PAS
Lactococcus lactis IBB3411	indicator strain	IBB PAS
Lactococcus lactis NZ9000	indicator strain, *pepN::nisRnisK* integrated into chromosome	MoBiTec[66]
Lactococcus lactis QU5 LMGT 3419	indicator strain	LMBT NMBU
Lactococcus lactis subsp. *cremoris* IBB3409	indicator strain	IBB PAS
Lactococcus lactis subsp. *lactis* IL1403	indicator strain, host strain	INRA[67]
Lactococcus lactis subsp. *lactis* IBB2955	indicator strain	IBB PAS
Lactococcus lactis subsp. *lactis* IBB3407	indicator strain	IBB PAS
Lactococcus raffinolactis IBB91	indicator strain	IBB PAS
Leuconostoc lactis IBB3446	indicator strain	IBB PAS
Leuconostoc mesenteroides IBB3442	indicator strain	IBB PAS
Leuconostoc mesenteroides IBB3443	indicator strain	IBB PAS
Listeria monocytogenes EGD-e LMGT 2604	indicator strain	LMGT NMBU[68]
Pediococcus acidilacti LMGT 2002	indicator strain	LMGT NMBU
Pediococcus parvulus IBB3448	indicator strain	IBB PAS
Pediococcus pentosaceus IBB3369	indicator strain	IBB PAS
Pseudomonas aeruginosa ATCC 9027	indicator strain	ATCC
Salomnella typhimurium TT622	indicator strain	[69]
Staphylococcus aureus ATCC 6538	indicator strain	ATCC
Staphylococcus caprae DSM-20608	indicator strain	DSMZ
Staphylococcus delphini DSM-20771	indicator strain	DSMZ
Staphylococcus epidermidis DSM-20044	indicator strain	DSMZ
Staphylococcus hyicus subsp. *chromogenes* DSM-20454	indicator strain	DSMZ
Staphylococcus intermedius DSM-20373	indicator strain	DSMZ
Staphylococcus lugdunesis DSM-4804	indicator strain	DSMZ
Staphylococcus pseudintermedius DSM-21284	indicator strain	DSMZ
Staphylococcus saprophyticus subsp. *saprophyticus* DSM-20229	indicator strain	DSMZ
Staphylococcus schleiferi subsp. *coagulans* DSM-6628	indicator strain	DSMZ
Streptococcus agalactiae IBB123	indicator strain	IBB PAS
Streptococcus agalactiae IBB130	indicator strain	IBB PAS
Streptococcus mitis IBB3449	indicator strain	IBB PAS
Streptococcus sobrinus IBB3450	indicator strain	IBB PAS
Streptococcus parauberis IBB272	indicator strain	IBB PAS
Lactococcus garvieae IBB3403 - spontaneous mutants		
PW202-PW204	GarQ-resistant, indicator strains	[12]
LGN1, LGN2, LGN4, LGN9	GarQ-resistant, indicator strains	[12]
MS1011-MS1014, MS1016-MS1018, MS1027-MS1029, MS1031-MS1033, MS1035-MS1036	GarA-resistant	This study
LGA2, LGA3, LGA5, LGA6, LGA13	GarA-resistant	This study
LGB1-LGB10	GarB-resistant	This study
LGC1, LGC3-LGC6, LGC8, LGC9, LGC11-LGC13, LC15, LGC16, LGC18-LGC20	GarC-resistant	This study
Lactococcus garvieae IBB3403 - other mutants		
MS1-MS3	strains after pGhost9::IS*S1* integration into chromosome	This study
B548a	strain with *manABCD* deletion	This study
B549a	strain with *manCD* deletion	This study
B550a	strain with *manC* deletion	This study
B551a	strain with *manD* deletion	This study
Lactococcus garvieae IBB3403 - other strains		
:pGh9::IS*S1*	strain carrying pGhost9::IS*S1*	This study
:pGh9:*manABCD*	strain carrying pGhost9 with *manABCD* deletion	This study
:pGh9:Δ*manCD*	strain carrying pGhost9 with *manCD* deletion	This study
:pGh9:Δ*manC*	strain carrying pGhost9 with *manC* deletion	This study
:pGh9:Δ*manD*	strain carrying pGhost9 with *manD* deletion	This study
B552a	B548a strain carrying pNZ9530	This study

Lactococcus lactis IL1403 strains with deleted *ptnABCD*		
B464	strain with *ptn*ABCD deletion	LMBT NMBU[4]
B488	B464 carrying pNZ9530	LMBT NMBU[4]
B520	B488 carrying pNZ8037	LMBT NMBU[4]
B557a	B488 carrying pNZ8037 with *manABCD*	This study
B558a	B488 carrying pNZ8037 with *manCD*	This study
B559a	B488 carrying pNZ8037 with *manC*	This study
B560a	B488 carrying pNZ8037 with *manD*	This study
B561a	B488 carrying pNZ8037 with *manCD* without γ+	This study
Lactococcus lactis NZ9000		
B562a	strain carrying pNZ8037	This study
B563a	strain carrying pNZ8037 with *manC*	This study
B564a	strain carrying pNZ8037 with *manD*	This study
Escherichia coli EC1000		
:pGh9::IS*S1*	strain carrying pGhost9::IS*S1*	This study
MS1 - MS3	strains carrying pGhost9::IS*S1* with part of *manABCD*	This study
:pGEMT:Δ*manABCD*	strain carrying pGEMT with *man* operon deletion	This study
:pGEMT:Δ*manCD*	strain carrying pGEMT with *manCD* deletion	This study
:pGEMT:Δ*manC*	strain carrying pGEMT with *manC* deletion	This study
:pGEMT:Δ*manD*	strain carrying pGEMT with *manD* deletion	This study
:pGh9:Δ*manABCD*	strain carrying pGhost9 with *man* operon deletion	This study
:pGh9:Δ*manCD*	strain carrying pGhost9 with *manCD* deletion	This study
:pGh9:Δ*manC*	strain carrying pGhost9 with *manC* deletion	This study
:pGh9:Δ*manD*	strain carrying pGhost9 with *manD* deletion	This study
:pNZ8037:*manABCD*	strain carrying pNZ8037 with *man* operon	This study
:pNZ8037:*manCD*	strain carrying pNZ8037 with *manCD*	This study
:pNZ8037:*manC*	strain carrying pNZ8037 with *man*C	This study
:pNZ8037:*manD*	strain carrying pNZ8037 with *manD*	This study
:pNZ8037:*manCD*Δ γ+	strain carrying pNZ8037 with *manCD* without γ+	This study
Plasmids		
pGhost9::IS*SI*	Em^r, *repA*(Ts), IS*S1*	[32]
pGhost9	Em^r, *repA*(Ts)	[32]
pGEMT	Amp^r, M13*ori*, linear T-overhang vector	Promega
pNZ9530	Em^r, carrying nisin-regulatory *nisRK* genes	[34]
pNZ8037	Cam^r,nisin regulated epression system containing nisin-responsive promoter	[33]
Primers	DNA sequence (5'->3'), restriction site	
pIS*S1Eco*/pGh9	CTAAAATAGACTTATCAG/GACAGCTTCCAAGGAGC	
uni/pIS*S1Hind*	GTAAAACGACGGCCAGT/AGCTTAAGAACAAGAAGG	
*manC*for/rev	CGTGATCTCGGCGTTA/TAACGCTCAAGCGTGTG	
*manD*for/rev	CGCTCTTATCTACCTC/GCCAATTTAGTGCTCCTAAC	
1224/1233	CGCCAGGGTTTTCCCAGTCACGA/AGCGGATAACAATTTCACACAGG	
pGhfor/rev	TGTAAAACGACGGCCAGTG/AGTACCGTTACTTATGAGC	
pNZ8037for/rev	CGATAACGCGAGCATA/GCTCAAGGGCTTTTACG	
*manABCD*UPfor/rev	GCTTTCATGGCCTTATAG/GAGAATTCCGTGGCTCGCAATAAC	
*manABCD*DNfor/rev	GAGAATTCACCACTGCTGGTATTG/CGTCGTTATGGGACTA	
*manCD*UPfor/rev	TGCACGTATCGACTCAC/GAGAATTCCAAGTCCAGCTAAGAAC	
*manC*DNfor/rev	GAGAATTCCGGCGATATTCTCAAC/GGACCAGTGAAGCTGATG	
*manD*UPfor/rev	GCCGCTATCTTGTTGG/GAGAATTCTAACGCTCAAGCGTGTG	
*manABCD*for/rev	CCGTGGTTTGCTCTAC/TGCTGCTGCGATGAGT	
*complman*for/rev	ATGCCCATGGATGATCTTCGGTGAGCAAGAG/ATGCCTCGAGTTATTAAGCAAGACCTGCAACGT	
*complCD*for	TAGCCCATGGATGAGTATTATTTCAATCATTTTAG	
*complC*rev	TAGCCTCGAGTTAATAATCGTTGAGAATATCG	
*complD*for	TAGCCCATGGATGGAAAATACAAATAAAAAGTTTAG	
γ+for/rev	ACAGTTGATGGTTCTA/TTTATCGATCCATTC	

* Restriction sites are underlined (EcoRI; NcoI; XhoI).

* Bacterial strains derived from the Regional Strains and Plasmids Collection of the Institute of Biochemistry and Biophysics, Warsaw, Poland (IBB PAS), from the Pure Cultures Collection of the Institute of Fermentation Technology and Microbiology, Technical University of Lodz, Poland (LOCK), from the dietary supplement Dicoflor, Vitis Pharma, Poland (Dicoflor), the collection of the Laboratory of Microbial Gene Technology, Department of Chemistry, Biotechnology and Food Science, Norwegian University of Life Sciences, Ås, Norway (LMGT NMBU), the Collection of Microorganisms and Cell Cultures, Germany (DSMZ), the MoBiTec GmbH, Germany (MoBiTec), the collection of the National Institute for Agricultural Research, France (INRA), the American Type Culture Collection (ATCC), and obtained in this study.

Table S2. Inhibitory spectrum of GarA, GarB and GarC. "–" indicates that no inhibition zone was observed, "+" indicates a wide, clear inhibition zone while +/- indicates a minimal, vague inhibition zone observed only at high concentration of bacteriocin (1 mg/ml).

Indicator strain	GarA	GarB	GarC
Bacillus cereus IBB3390	-	-	-
Bacillus subtilis BSB1	-	-	-
Campylobacter jejuni 12	-	-	-
Campylobacter jejuni 480	-	-	-
Campylobacter jejuni 81176	-	-	-
Campylobacter coli 23/1	-	-	-
Candida albicans CAI-4	-	-	-
Carnobacterium maltaromaticum IBB3447	+/-	-	-
Enterococcus durans IBB3441	+/-	-	-
Enterococcus faecalis IBB3439	-	-	-
Enterococcus faecalis IBB3444	-	-	-
Enterococcus faecalis LMGT 2003	-	-	-
Enterococcus faecium LMGT 2783	+/-	-	-
Enterococcus faecium LMGT 2787	+/-	-	-
Lactobacillus casei IBB3418	-	-	-
Lactobacillus casei IBB3427	-	-	-
Lactobacillus casei LOCK 0919	-	-	-
Lactobacillus casei/paracasei IBB3423	-	-	-
Lactobacillus casei/paracasei IBB3425	-	-	-
Lactobacillus casei/paracasei IBB3426	-	-	-
Lactobacillus casei/paracasei IBB3428	-	-	-
Lactobacillus paracasei IBB3424	-	-	-
Lactobacillus johnsonii IBB3155	+/-	-	-
Lactobacillus kunkeei AH1	-	-	-
Lactobacillus kunkeei AH38	-	-	-
Lactobacillus kunkeei AH119	-	-	-
Lactobacillus paraplantarum IBB3438	+/-	-	+/-
Lactobacillus plantarum NC8	-	-	+/-
Lactobacillus plantarum WCSF1	-	-	-
Lactobacillus plantarum IBB3036	-	-	-
Lactobacillus plantarum IBB3433	+/-	-	+/-
Lactobacillus plantarum IBB3436	+/-	-	-
Lactobacillus plantarum subsp. *plantarum* IBB3434	+/-	-	-
Lactobacillus rhamnosus IBB3429	-	-	-
Lactobacillus rhamnosus LOCK 0900	+/-	-	-
Lactobacillus rhamnosus LOCK 0908	+/-	-	-
Lactobacillus rhamnosus GG	-	-	-
Lactobacillus salivarius IBB3154	+/-	-	+/-
Lactococcus garvieae IBB3403	+	+	+
Lactococcus garvieae IBB66	+	+	+
Lactococcus lactis IBB3404	+/-	-	+
Lactococcus lactis IBB3411	+/-	-	+
Lactococcus lactis NZ9000	+/-	-	+
Lactococcus lactis QU5 LMGT 3419	+/-	-	+
Lactococcus lactis subsp. *cremoris* IBB3409	+/-	-	+
Lactococcus lactis subsp. *lactis* IL1403	+/-	-	+
Lactococcus lactis subsp. *lactis* IBB2955	+/-	-	+
Lactococcus lactis subsp. *lactis* IBB3407	+/-	-	+
Lactococcus raffinolactis IBB91	-	-	+
Leuconostoc lactis IBB3446	+/-	-	-
Leuconostoc mesenteroides IBB3442	-	-	-
Leuconostoc mesenteroides IBB3443	-	-	+/-
Listeria monocytogenes EGD-e LMGT 2604	-	-	-
Pediococcus acidilacti LMGT 2002	+/-	-	-
Pediococcus parvulus IBB3448	-	-	-
Pediococcus pentosaceus IBB3369	-	-	-
Pseudomonas aeruginosa ATCC 9027	-	-	-
Salomnella typhimurium TT622	-	-	-
Staphylococcus aureus ATCC 6538	-	-	-
Staphylococcus caprae DSM-20608	-	-	-
Staphylococcus delphini DSM-20771	-	-	-

Staphylococcus epidermidis DSM-20044	-	-	-
Staphylococcus hyicus subsp. *chromogenes* DSM-20454	-	-	-
Staphylococcus intermedius DSM-20373	-	-	-
Staphylococcus lugdunesis DSM-4804	-	-	-
Staphylococcus pseudintermedius DSM-21284	-	-	-
Staphylococcus saprophyticus subsp. *saprophyticus* DSM-20229	-	-	-
Staphylococcus schleiferi subsp. coagulans DSM-6628	-	-	-
Streptococcus agalactiae IBB123	-	-	-
Streptococcus agalactiae IBB130	-	-	-
Streptococcus mitis IBB3449	-	-	-
Streptococcus sobrinus IBB3450	-	-	-
Streptococcus parauberis IBB272	-	-	-
